# Association between PM_2.5_ and skin redness features in Taiwan

**DOI:** 10.1371/journal.pgph.0004357

**Published:** 2025-03-12

**Authors:** Fu-Yu Chan, Chia-Pin Chio, Tzu-Hsuen Yuan, Shu-Fang Shih, Chao-Jen Shih, Chang-Chuan Chan, Yaung-Chuan Lee, Chie-Chien Tseng

**Affiliations:** 1 Skin Health Promotion Research Centre, UNIYU Biotech Corporation, Taipei City, Taiwan; 2 Department of Health Promotion and Health Education, College of Education, National Taiwan Normal University, Taipei, Taiwan; 3 Department of Medical Research, Tung’ Taichung Metro Harbor Hospital, Taichung, Taiwan; 4 Department of Health and Welfare, College of City Management, University of Taipei, Taipei City, Taiwan; 5 Department of Health Administration, College of Health Professions, Virginia Commonwealth University, Richmond, Virginia, United States of America; 6 Institute of Health Policy and Management, College of Public Health, National Taiwan University, Taipei, Taiwan; 7 Dermatologic Clinic of Shih Chao-Jen, Taipei, Taiwan; 8 Institute of Environmental and Occupational Health Sciences, College of Public Health, National Taiwan University, Taipei, Taiwan,; 9 Department of Cosmetology and Fashion Design, Ching Kuo Institute of Management & Health, Keelung, Taiwan; PLOS: Public Library of Science, UNITED STATES OF AMERICA

## Abstract

Air pollution, particularly fine particulate matter (PM2.5), has been associated with various health issues, but its effects on skin health, specifically skin redness, remain underexplored. This study aims to examine the relationship between PM2.5 exposure and skin redness, with a focus on the role of sebum production in different age groups. A total of 472 participants from two communities in Taiwan in two age groups (20–59 years, n=240; over 60 years, n=232) were included in the study. PM2.5 exposure levels were estimated using land use regression models based on participants’ residential addresses. Skin redness area was assessed using the VISIA Imaging System. Linear regression analyses were conducted to examine the association between PM2.5 and redness area, adjusting for demographic, lifestyle, and ultraviolet exposure. Results showed a significant positive association between PM2.5 levels and redness area in both age groups. In the 20-59 age group, each unit increase in PM2.5 corresponded to a 1.70-unit increase in redness area (95% CI: 0.32 – 3.07, p < 0.01), while in the over-60 group, the increase was 2.63 units (95% CI: 1.19 – 4.08, p < 0.001). Additionally, porphyrins showed a positive association with redness area among the 20-59 age group (p < 0.05), while no significant association was found in the over-60 group. This study suggests a linkage between PM2.5 exposure and skin redness area, indicating that air pollution may be a contributing factor to skin health issues. The findings suggest that the interaction between lipophilic and carcinogenic substances in PM2.5 and porphyrins could elevate redness area levels and potentially increase the risk of chronic skin conditions and skin cancer.

## Introduction

The World Health Organization (WHO) global air quality guidelines (2021) recommend that the annual Air Quality Guideline (AQG) level for particulate matter with an aerodynamic diameter of less than 2.5 µm (PM2.5) be set at 5 µg/m³, while the short-term (24-hour) AQG level is 15 µg/m³. These guidelines are based on assessments of both all-cause non-accidental and cause-specific mortality risks [1]. According to the Global Burden of Disease (GBD) 2018 statistics, the mortality rate attributed to particulate matter pollution for individuals aged 70 and above in Taiwan was reported at 366.24 per 100,000 [2]. Additionally, a study by Chio et al. found that PM2.5 emissions from coal-fired power plants in Taiwan were responsible for 359.6 adult deaths and 124.4 premature deaths annually, with the maximum affected distances determined to be 272 kilometers for coal power plants and 157 kilometers for cogeneration power plants [3].

Between 2010 and 2013, a series of cohort studies on air pollution and environmental health revealed the presence of numerous toxic air pollutants emitted from industrial parks in the coastal areas of central Taiwan. These pollutants included highly lipophilic and carcinogenic volatile organic substances, with the highest concentrations of 52 major hazardous air pollutant components within a 10-kilometer radius of the industrial areas, including benzene, ethylene, propylene, 1-hydroxypyrene (1-OHP), polycyclic representatives, and heavy metals. In residents’ urine, biomarkers for pollutants such as vanadium (V), strontium (Sr), arsenic (As), lead (Pb), mercury (Hg), and thallium (Tl) were substantially higher compared to levels within this range [4]. PM_2.5_ possess a size smaller than skin pores (<100 μm) and can penetrate the skin through hair follicles to depths of 200-500 μm, reaching deeper skin layers [5]. These particles also contain polar compounds that increase the permeability of the stratum corneum and enter the basal layer through the hair follicles. This process induces aryl hydrocarbon receptors (AhR), leading to skin discomfort (e.g., tingling, itching, burning sensation, dryness, erythema, desquamation, papules, or scales), accelerated skin aging, disruption of the skin barrier function [6–14], and an increased risk of skin cancer [15].

Given these known effects of PM2.5 on deeper skin layers, its association with visible skin conditions, such as skin redness, is of particular concern. Skin redness, or erythema, is a visible indication of underlying inflammation or irritation and is commonly regarded as a sign of compromised skin health [16,17]. It can result from various factors, such as exposure to environmental pollutants like PM2.5, ultraviolet (UV) radiation, or other irritants that disrupt the skin’s protective barrier [14,18,19]. Notably, UV radiation not only directly induces skin redness through mechanisms such as oxidative stress and inflammation but may also interact with PM2.5 to exacerbate skin damage [14,20]. Studies suggest that UV exposure can alter the skin’s permeability and barrier function, potentially enhancing the penetration and impact of lipophilic pollutants like PM2.5, leading to heightened inflammatory responses and more severe skin conditions [21]. In the context of skin health, skin redness is not merely a cosmetic concern but a significant marker of dermal stress and dysfunction, reflecting the skin’s inability to maintain its barrier function effectively [20,22]. Consequently, addressing skin redness is critical for preventing further skin damage and ensuring overall skin health, particularly in populations exposed to high levels of environmental pollutants [20].

PM2.5 has been reported to impact cholesterol metabolism and increase epidermal cholesterol levels [23–25]. Human sebum, which exhibits mild antibacterial activity, acts as a natural protective barrier. Sebum production on the skin surface helps maintain smooth and supple skin, except in children, whose sebaceous glands are not fully developed. Almost all cholesterol, cholesterol esters, triglycerides, diglycerides, and free fatty acids originate from sebum, and their production significantly varies with age [5]. Sebum production is mainly concentrated on the face and scalp in adults, and its levels vary depending on age. For instance, sebum production on the forehead and cheeks in adult males is 160 μg/cm² and 104 μg/cm², respectively. In younger age groups (20-39 years), sebum production levels are higher but tend to decrease with age, particularly in females, as shown by the lower levels in individuals aged 60-79 years [5]. Age-related variations in sebum production may exacerbate skin conditions caused by environmental exposures, such as PM2.5. The lipophilic nature of PM2.5 allows it to interact with sebum, potentially leading to oxidative stress and inflammation, contributing to skin redness. Understanding this interaction is crucial for developing strategies to protect vulnerable populations, especially older adults with reduced sebum production, from the harmful effects of air pollution on skin health.

Although it is well established that PM2.5 contains carcinogenic lipophilic substances that readily interact with sebum, there is limited research on the specific adverse effects of these pollutants on skin health. PM2.5 is known to penetrate both the epidermis and dermis, potentially through hair follicles, leading to deeper tissue exposure. However, despite these established mechanisms of penetration, few studies have explored the direct impact of PM2.5 on skin conditions such as redness, particularly with regard to how lipophilic pollutants interact with skin and sebum across different age groups. To address this knowledge gap, this study aims to provide a comprehensive understanding of the effects of PM2.5 air pollution on skin health, with a specific focus on the relationship between lipophilic pollutants (i.e., PM2.5) and skin redness area, and how this relationship varies across age groups. Through this research, we seek to offer valuable insights into the public health implications of air pollution and provide guidance on protective measures to mitigate its harmful effects on skin health.

## Methods

### Study design

Informed by previous research in Taiwan, this study focuses on the impact of PM2.5 emissions, notably from Taiwan’s coal-fired power plants, which are linked to 359.6 adult and 124.4 premature deaths per year. These plants influence areas up to 272 kilometers from coal power stations and 157 kilometers from cogeneration plants [3]. This study employed an observational cross-sectional design to investigate the association between PM2.5 exposure and skin redness. The rationale for this design was to examine the relationship between environmental pollutants and skin health within a defined population at a single point in time. The study focused on regions in Taiwan with known high levels of PM2.5 exposure. Additionally, research conducted from 2010 to 2013 highlighted the significant emissions of toxic air pollutants, including carcinogenic and lipophilic substances, from industrial parks in central Taiwan. Notably, within 10 kilometers of these parks, there is a high concentration of 52 major hazardous air pollutants [4]. To understand the effects of these pollutants on skin health, this cross-sectional study randomly selected individuals who have resided in central and northern Taiwan for more than one year.

### Study participants

Participants’ eligibility was initially confirmed based on the inclusion and exclusion criteria during recruitment in various communities. Inclusion criteria required participants to have resided in central or northern Taiwan for at least one year. Participants also needed to complete the skin health questionnaire and agree to undergo a VISIA Imaging System skin analysis. Exclusion criteria included individuals diagnosed with chronic skin diseases, such as eczema or psoriasis, as these conditions may independently affect skin redness. Additionally, participants undergoing dermatological treatments, such as laser therapy, were excluded to minimize confounding factors in skin health measurements. The recruitment period ran from August 1st, 2018, to November 30th, 2018. A barcode link was then provided to access the skin health questionnaire website. Participants were informed during the questionnaire process that they could withdraw consent at any time without providing a reason. Community residents who completed the skin health questionnaire and agreed to undergo skin examinations were provided with skin examination devices at a designated venue. Local facial photographs were taken, followed by skin analysis. All photographs were de-identified to ensure the protection of personal data.

### Informed consent and ethical approval

This study was conducted in accordance with the World Medical Association Declaration of Helsinki guidelines. This study followed an informed consent process, complied with ethical regulations, and was approved by the Human Research Ethics Committee of Taipei City University (IRM-2021-071). All the participants provided informed consent under ethical regulations. The information regarding the ethical, cultural, and scientific considerations specific to inclusivity in global research is included in the Supporting Information (S1 Checklist).

### Device for measuring skin redness area and sebum production

The VISIA Imaging System (Canfield, NJ, USA) and VISIA skin analysis software capture high-resolution facial images (18 million pixels) with accurate front-facing 0-degree and left/right side-facing 33-degree images. This instrument uses unique flash modes, such as UV light at 365nm and RBX cross-polarized light, each tailored to examine specific skin features. Leveraging photobiology principles, these light sources deeply penetrate the skin, revealing conditions invisible to the naked eye. In this research, participants’ skin redness was analyzed using RBX polarized light technology, which detects blood vessels and hemoglobin in deeper skin layers, helping identify issues like acne, inflammation, rosacea, and vascular lesions. The feature count method was used to quantify the number of distinct occurrences of these features, regardless of their size or intensity, proving useful in monitoring treatment efficacy.

Sebum production was determined by measuring porphyrin levels under UV light. Porphyrins, as an indicator of sebum production, are produced by acne-causing bacteria and fluoresce under UV light. Elevated porphyrin levels indicate higher oil content, potentially leading to increased acne bacterial growth. The feature count also recorded the number of discrete porphyrin occurrences. This technique, which includes both instrument and software analysis, is widely used in academic research to track skin porphyrins. The system is highly effective in evaluating skin sebum production and redness, converting these assessments into measurable scores. It leverages a vast reference database with data from hundreds of thousands of individuals, factoring in gender, age, and specific skin features [26–30].

The skin health assessments were conducted by the Skin Health Promotion Research Centre (SHPR). Certified personnel (TAF-QM107022-C-23) operated the instruments, following ISO17025 standards. It is important to note that the researchers involved in this study were certified to operate the measurement device, and during the testing phase, the device was operated in strict compliance with ISO17025 specifications, ensuring the reliability and accuracy of the data collection process. The data provided by SHPR were fully anonymized, ensuring no personal information could be identified.

### Questionnaire on skin health, lifestyle, and environmental exposure

The questionnaire was designed in our previous study [31]. Information includes basic demographic data, lifestyle profiles, and environmental exposure-related factors. The study variables are described as follows:

1. Basic information: included gender, residential (or work) address, whether the participants had resided in the current address for at least one year, and age. The residential address was converted to spatial coordinates to calculate outdoor air pollution exposure.
2. Lifestyle profile: Mask-wearing habit refers to whether the participants have the habit of wearing a mask, and the options include never, occasionally, sometimes, often, and always. In this study, mask-wearing were classified as yes or no.
3. Environmental exposure:
  (1) Chemicals: Use of facial protection products refers to whether the participants have a habit of using sunscreen lotion (or sunblock lotion/foundation with skin protection factor), and the options include never, occasionally, sometimes, often, and always. In this study, data on sunscreen use were classified as yes or no.
  (2) UV Exposure Risk Assessment: The UV exposure risk was evaluated using a questionnaire based on Fitzpatrick grading [32], assessing participants’ outdoor activities and intentional sun exposure. Participants were asked three questions: whether they worked outdoors, including farming activities; whether they frequently engaged in outdoor activities; and whether they intentionally exposed themselves to sunlight for more than four hours. Participants who answered positively to two or more of these questions were classified as having a high UV exposure risk, while others were classified as low UV exposure risk.

### Estimation of the exposure to air pollution of the participants

To assess participants’ pre-existing exposure to air pollution prior to their involvement in this study, land use regression (LUR) models were employed. Lee et al. [33] previously used this method to evaluate the exposure of participants in urban Taipei in 2008. In this study, the model developed by Lee et al. was applied to estimate the long-term PM2.5 exposure of participants in the Taipei metropolitan area, using long-term monitoring data from the nearest Taiwan Environmental Protection Administration (TEPA) air quality monitoring station (AQMS), adjusted to align with the study’s time frame. Exposure was estimated based on participants’ residential addresses, and the annual average air pollutant concentrations for 2018 were calculated.

For participants over 60 years old residing in Changhua, additional LUR models specific to Changhua County [27,28] were applied. PM2.5 samples in Changhua County were measured and modeled in 2014, with revisions in 2017. To improve the assessment of PM2.5 exposure from major stationary sources, a Gaussian trajectory transfer-coefficient model system (GTx model) was used, which accounts for the transport and dispersion of pollutants. PM2.5 exposure in Changhua Community participants for 2018 was adjusted using continuous data from 2014 to 2018 collected by the Changhua AQMS. The analytical steps for this method are as follows:

1. Obtain the spatial coordinates of the residential and/or work locations of each participant from two selected communities, where they had resided for more than one year.
2. Using the LUR models, predict variables related to long-term exposure to PM2.5, such as road area, residential area, industrial area, commercial area, construction site area, river area, urban green space, and natural land area.
3. Conduct geospatial analysis to obtain values for these variables around each participant’s spatial coordinates.
4. Apply the LUR models specific to the Taipei metropolitan area and the Changhua suburban area to assess the annual average PM2.5 exposure for each participant in 2018. The regression coefficients of the model parameters were provided in previous studies by Lee et al. [33].

The coefficients from Chan et al. [31] were derived by sampling various air pollutants (including PM2.5) and collecting relevant land-use characteristics near the sampling points. The relationship between pollutant concentrations and environmental characteristics was established through multivariate linear regression analysis. Data used to construct the model were collected from 2014 to 2015, representing conditions during that period. This study assumes that the environmental characteristics (land-use conditions) around participants’ locations (aged 20-59 years) remained relatively stable between 2014 and 2018. Long-term air pollutant trends from 2014 to 2018 were adjusted using data from TEPA’s air quality monitoring stations, with model coefficients assumed to remain unchanged.

### Statistical analysis

We first reported frequencies and percentages to present information collected by the questionnaire on basic demographic characteristics, health behaviors, and environmental exposures. PM2.5 exposure and skin health indicators were summarized using mean values and standard deviations. Based on literature [5], participants were stratified into two age groups: 20–59 years and 60 years or above to account for age differences in sebum production. We used Pearson Chi-square tests and independent sample t-tests to examine differences in participant characteristics between the two age groups. To examine the association between PM2.5 exposure and redness area across the two age groups, we conducted linear regression models, controlling for gender, porphyrins, mask-wearing, sunscreen usage, and high UV exposure risk. Mask-wearing was included as a control variable due to its potential role in reducing direct skin exposure to PM2.5, sunscreen use was included to account for the confounding effects of UV protection on skin redness, and high UV exposure risk was included to reflect its potential contribution to skin damage and redness. All statistical analyses were performed using SPSS version 23 software, and all tests were two-tailed.

## Results

### Participant characteristics

A total of 472 participants were included in the study, of which 69.1% were female and 50.8% were aged 20-59 years, while 49.2% were over 60 years (Table 1). Most participants resided in Taipei (91.1%). In terms of lifestyle, 48.9% reported having a mask-wearing habit, and 36.9% used sunscreen regularly. Additionally, 5.3% of participants were categorized as having a high UV exposure risk. The average PM2.5 exposure among participants was 21.9 μg/m³ (SD = 6.0), the mean skin redness area score was 191.4 (SD = 75.8), and the mean porphyrins was 1175.3 (SD = 1036.5).

**Table 1:** Participant characteristics and environmental exposure by age groups.

Variables	Total (n = 472)		20-59 years (n = 240)		Over 60 years (n = 232)		*p*-value
	n	%	n	%	n	%	
**Gender**							0.025
Female	326	69.1	177	73.8	149	64.2	
Male	146	30.9	63	26.3	83	35.8	
**Age Group**							
20-59 years	240	50.8					
Over 60 years	232	49.2					
**Area**							0.001
Taixi Village	42	8.9	11	4.6	31	13.4	
Taipei Community	430	91.1	229	95.4	201	86.6	
**Wearing mask habit**							0.001
No	241	51.1	105	43.8	136	58.6	
Yes	231	48.9	135	56.3	96	41.4	
**Using sunscreen habit**							0.0001
No	298	63.1	132	55.0	166	71.6	
Yes	174	36.9	108	45.0	66	28.4	
**Ultraviolet exposure risk**							0.906
Low	447	94.7	227	94.6	220	94.8	
High	25	5.3	13	5.4	12	5.2	
**PM2.5 exposure** (mean, SD)	21.9	6.0	21.5	6.3	22.3	5.7	0.162
**Skin redness area** (mean, SD)	191.4	75.8	162.7	73.0	220.9	66.9	0.0001
**Sebum production** (mean, SD)	1175.3	1036.5	1207.7	1034.3	1141.8	1040.0	0.490

P-values were calculated by Pearson χ2 test and independent sample t-tests.

In the bivariate analysis of age and characteristics, a higher proportion of females was observed in the 20-59 age group (χ² = 5.010, p =.025), and a higher percentage of participants in this age group resided in Taipei (χ² = 11.215, p <.001). Additionally, participants aged 20-59 had a higher likelihood of reporting mask usage habits (χ² = 10.439, p =.001) and sunscreen usage (χ² = 13.886, p <.001). In contrast, there was no significant relationship between age and UV exposure risk (χ² =.014, p =.906), indicating similar levels of UV exposure risk across age groups. Moreover, no significant differences were observed in PM2.5 exposure and porphyrins between the two age groups. However, participants over 60 exhibited higher mean levels of redness area compared to those aged 20-59 (p <.001), indicating age-related differences in redness area.

### Association between air pollutants and skin redness area

Table 2 presents the results of the linear regression analysis, which shows a significant positive association between air pollution and skin redness area after controlling for various covariates. Specifically, in the 20-59 age group, each unit increase in PM2.5 was associated with a 1.70-unit increase in redness area (95% CI = 0.32–3.07, p < 0.01), while in the over-60 age group, each unit increase in PM2.5 was associated with a 2.63-unit increase in redness area (95% CI = 1.19–4.08, p < 0.001). Notably, porphyrins were positively associated with redness area in the 20-59 age group (p < 0.05), whereas no significant relationship was found in the over-60 group. Mask-wearing, sunscreen usage, and high UV exposure risk were not significantly associated with redness area in either age group. However, in both age groups, males exhibited higher levels of redness area compared to females.

**Table 2:** Associations of air pollutants with skin redness area.

	20-59 years (n = 240)			Over 60 years (n = 232)		
Variables	b	95% CI		b	95% CI	
PM_2.5_	1.70**	0.32	3.07	2.63***	1.19	4.08
Porphyrins	0.01*	0.00	0.02	0.01	0.00	0.02
Wearing Mask	-9.20	-27.00	8.59	-7.52	-23.69	8.64
Sunscreen Usage	8.20	-10.64	27.04	-13.06	-31.76	5.64
High UV Exposure Risk	7.91	-30.25	46.07	20.54	-16.05	57.12
Male	45.97***	19.38	72.57	24.69*	0.82	48.55

*Note*. *p<0.05, **p<0.01, ***p<0.001

The linear regression model was employed to understand the relationship between environmental factors (like PM_2.5_) and skin redness, accounting for other variables such as porphyrins and lifestyle habits.

## Discussion

Our study provides valuable insights into the association between PM2.5 air pollution exposure and skin redness area. The findings suggest that higher levels of PM2.5 are consistently associated with an increase in redness area across different age groups, even after accounting for factors such as lifestyle habits, environmental exposure, and skin characteristics. Sebum production also played a role in skin redness area, particularly among younger participants. Notably, protective behaviors like mask-wearing and sunscreen use did not significantly associate with redness area, indicating the persistence of PM2.5’s association with skin health.

These findings align with prior studies showing that PM2.5 can penetrate the skin through hair follicles, reaching depths of 200-500 µm and triggering processes that can lead to skin issues like erythema, discomfort, and an increased risk of skin cancer [5,6,15,34]. Despite consistent PM2.5 exposure levels across both age groups, our findings on porphyrins align with broader environmental studies. While traditional research suggests that sebum production decreases with age [5], our data indicate that participants over 60 exhibit sebum production levels similar to those in the 20-59 age group, which contrasts with research showing a marked decrease in sebum production among older adults [23]. The similarity in sebum production across age groups could suggest the influence of external pollutants, including lipophilic substances, which have been shown to interact with sebum and increase the risk of skin conditions [5,23–25].

The ability of PM2.5 particles to penetrate the skin, particularly through hair follicles, and to affect deeper skin layers is a significant concern [18,19]. These particles, smaller than 100 μm, can infiltrate the epidermis and, through increased permeability, enter deeper skin layers, triggering a cascade of skin-related issues such as discomfort, disrupted barrier function, and increased risk of skin cancer [6–15]. The role of lipophilic and carcinogenic substances in PM2.5, as identified in cohort studies conducted in Taiwan’s coastal industrial areas, supports this conclusion [4]. These studies reported elevated levels of hazardous air pollutants and heavy metals in residents’ urine, reflecting the environmental burden in these regions [4,35].

In our analysis, we observed an association between porphyrins and redness areas in the 20–59 age group, suggesting a link between PM2.5-related skin redness and the physiological characteristics of younger adults [5,6]. This relationship may be attributed to higher sebum production levels in this age group, which provide an ideal medium for pollutant adhesion and absorption [5–7]. Elevated sebum levels facilitate the skin’s uptake of lipophilic substances in PM2.5, exacerbating inflammatory responses and increasing redness areas [23–25]. The interaction between PM2.5 particles and sebum may induce oxidative stress and the production of inflammatory markers, contributing to skin redness [23–25]. These findings underscore the role of porphyrins as mediators in the skin’s response to pollutants in younger individuals. Our findings achieve the study’s objective of providing a comprehensive understanding of the effects of PM2.5 air pollution on skin health, especially regarding the interaction between lipophilic pollutants and sebum production.

This study’s limitations are notably characterized by its geographical scope, focusing primarily on central and northern Taiwan. This regional concentration might not fully capture the diverse environmental conditions and pollution levels present in other areas, potentially limiting the broader applicability and generalizability of the findings. In addition, the sample size from the Taixi region was relatively small, which undermines the reliability of comparisons between regions. Incorporating more regions in future research could provide a more comprehensive understanding of the impact of PM2.5 on skin health, considering the varied industrial and environmental landscapes across the entire island. Another limitation is the gender imbalance in the study, with 70% of participants are female. This uneven distribution limits the validity of gender-based comparisons, particularly in evaluating differences in skin redness or porphyrins. Moreover, cosmetic practices, which were not accounted for in this study, could also confound the findings. Differences in skincare habits between genders, for example, might partly explain the observed disparities in skin redness or porphyrins. Furthermore, future studies could account for participants’ daily outdoor habits, as individuals living at the same address may have different PM2.5 exposure levels based on their time spent outdoors. Finally, there is the potential for participant withdrawal bias. By allowing participants to withdraw from the study at any time, in line with ethical standards, the study may inadvertently introduce a selection bias. This could affect the study’s outcomes, particularly if those who withdrew had unique characteristics or experiences directly related to the study’s focus areas, such as specific skin health concerns or discomfort during the examination process.

## Conclusion

This study suggests an association between PM2.5 levels and skin redness area across different age groups. In both individuals over 60 years old and those aged 20–59, an increase in PM2.5 levels is linked to an elevated risk of skin redness. Furthermore, higher sebum production is also associated with increased redness area, particularly in the younger age group. Based on these findings, it is recommended that public health authorities implement annual skin health screenings, using skin redness as a potential indicator of PM2.5 exposure to prompt further evaluation of the broader health impacts of air pollution. Additionally, promoting the use of scientifically tested and proven protective products, such as creams formulated to enhance the skin’s defense against PM2.5, could help reduce pollutant penetration and mitigate skin damage, particularly in populations exposed to high levels of air pollution.

## Supporting information

S1 Checklist Inclusivity in global research.
(DOCX)
